# Tiny molecules, mighty impacts: MicroRNAs in muscular dystrophies and myopathies

**DOI:** 10.1055/s-0046-1829138

**Published:** 2026-11-10

**Authors:** Ana Luiza Fabris-Moraes, Paula Fratini, Enzo Pellacani, David Feder, Alzira Alves de Siqueira Carvalho

**Affiliations:** 1 Centro Universitário Faculdade de Medicina do ABC, Faculdade de Medicina, Santo André SPBrazil.; 2 Centro Universitário Faculdade de Medicina do ABC, Departamento de Farmacologia, Santo André SPBrazil.; 3 Centro Universitário Faculdade de Medicina do ABC, Departamento de Neurociências, Serviço Neuromuscular, Santo André SPBrazil.

**Keywords:** MicroRNAs, Muscular Dystrophies, Muscular Diseases, Biomarkers

## Abstract

Microribonucleic acids (miRNAs) are small non-coding RNA molecules that regulate gene expression post-transcriptionally and play essential roles in muscle differentiation, regeneration, and metabolism. A specific subset, known as myomiRs, is predominantly expressed in muscle tissue and has been implicated in key molecular pathways underlying muscular dystrophies and myopathies. Due to their stability in biological fluids, myomiRs have emerged as non-invasive biomarkers. The present narrative review summarizes selected studies addressing myomiR expression patterns, functional roles, and translational implications in muscular dystrophies and myopathies, particularly Duchenne muscular dystrophy (DMD), myotonic dystrophy type 1 (DM1), limb-girdle muscular dystrophy (LGMD), McArdle disease, and myofibrillar myopathy (MFM), highlighting their biological relevance and clinical potential. The available evidence indicates that myomiRs such as miR-1, miR-133a/b, miR-206, miR-29b, miR-31, and miR-486 are differentially expressed across these disorders and are involved in processes including muscle degeneration, regeneration, fibrosis, and inflammation. In DMD, several myomiRs are upregulated, reflecting ongoing muscle damage and regenerative activity, whereas in DM1, downregulation of miR-1, miR-133a/b, and miR-29b contributes to impaired muscle maturation and fibrosis. In contrast, miR-206 appears increased as part of a regenerative response, whereas miR-223–3p may reflect inflammatory activity or muscle damage. Evidence for McArdle disease remains scarce, underscoring the need for further investigation. In conclusion, understanding miRNA signatures may facilitate earlier diagnosis, disease stratification, and the development of targeted therapies in muscular dystrophies and myopathies.

## INTRODUCTION

Microribonucleic acids (miRNAs) are a class of endogenous, small, single-stranded non-coding RNAs, typically 19 to 25 nucleotides in length, that do not encode proteins. In biological fluids, circulating miRNAs are found not only encapsulated within extracellular vesicles, such as exosomes, but also associated with RNA-binding proteins (e.g., Argonaute proteins) or lipoprotein complexes, which contribute to their remarkable stability. MicroRNAs play a central role in post-transcriptional gene regulation. They exert their function through incorporation into the RNA-induced silencing complex (RISC), where they guide the complex to target messenger RNAs (mRNAs). This interaction is primarily mediated by partial complementarity between the miRNA seed sequence (nucleotides 2–8 at the 5′ end) and sequences located in the 3′ untranslated region (3′ UTR) of target mRNAs, resulting in mRNA degradation and/or translational repression. In some cases, miRNAs may also interact with the 5′ UTR or coding regions, further modulating gene expression. 1 2

A growing body of evidence indicates that miRNAs are intricately involved in fundamental biological processes, including the regulation of cell proliferation, apoptosis, cellular differentiation, and hematopoiesis. Moreover, they have garnered increasing attention in the context of muscular dystrophies and myopathies, where they function as critical modulators of gene expression, influencing key pathways such as muscle regeneration, inflammation, and degeneration. Their potential utility as both biomarkers and therapeutic targets has sparked considerable interest, particularly in skeletal muscle disorders. Notably, the remarkable stability of miRNAs in biological fluids facilitates their reliable detection, underscoring their promise as minimally-invasive biomarkers across a range of pathological conditions, including muscular dystrophies and myopathies. 3 4 5 6 7

These disorders encompass a diverse set of genetic or acquired etiologies that affect muscle function, often resulting in progressive muscle weakness and motor dysfunction. Within this group, muscular dystrophies (MDs) stand out, such as Duchenne muscular dystrophy (DMD) and Becker muscular dystrophy (BMD), myotonic dystrophy type 1 (DM1), limb-girdle muscular dystrophy (LGMD), and congenital muscular dystrophy. This also applies to other metabolic and distal myopathies caused by pathogenic variants in genes responsible for regulating muscle function, which leads to the dysfunction of essential proteins for muscle integrity. 7

The aim of the current review is to summarize the current literature on the role of miRNAs in muscular dystrophies and myopathies, emphasizing their importance in diagnosis and treatment.

## MICRORIBONUCLEIC ACIDS (MiRNAs)

### The origin of miRNAs

MicroRNAs were discovered in 1993 in the nematode *Caenorhabditis elegans* , whose cel-lin-4 gene (MI0000002) regulates larval development by inhibiting the translation of cel-lin-14 mRNA without degrading it. 8 9

In 2000, another miRNA was identified in this organism, let-7, later recognized as the first miRNA conserved in humans (hsa-let-7, MI0000060). 10 11 Since then, thousands of miRNAs have been identified in different species and cataloged in the miRBase database. 12 13

MicroRNAs are transcribed primarily by RNA polymerase II as long primary transcripts (pri-miRNAs), which are characterized by a stem-loop (hairpin) structure, as well as a 5′ cap and a poly(A) tail. In the nucleus, the pri-miRNA is processed by the Microprocessor complex, composed of the RNase III enzyme Drosha and its cofactor DGCR8, generating a precursor miRNA (pre-miRNA) of approximately 70 nucleotides. The pre-miRNA is then exported to the cytoplasm via exportin-5 in a ran-GTP-dependent manner. In the cytoplasm, it undergoes further cleavage by the RNase III enzyme Dicer, producing a double-stranded miRNA duplex of approximately 22 nucleotides. One strand of this duplex, known as the guide strand, is preferentially incorporated into the RNA-induced silencing complex (RISC), which contains Argonaute (AGO) proteins as its core components. This strand will regulate gene expression by preventing the production of certain proteins. The opposite strand (passenger strand) is typically degraded 14 15 16 17 18 ( Figure 1 ).

**Figure 1:**
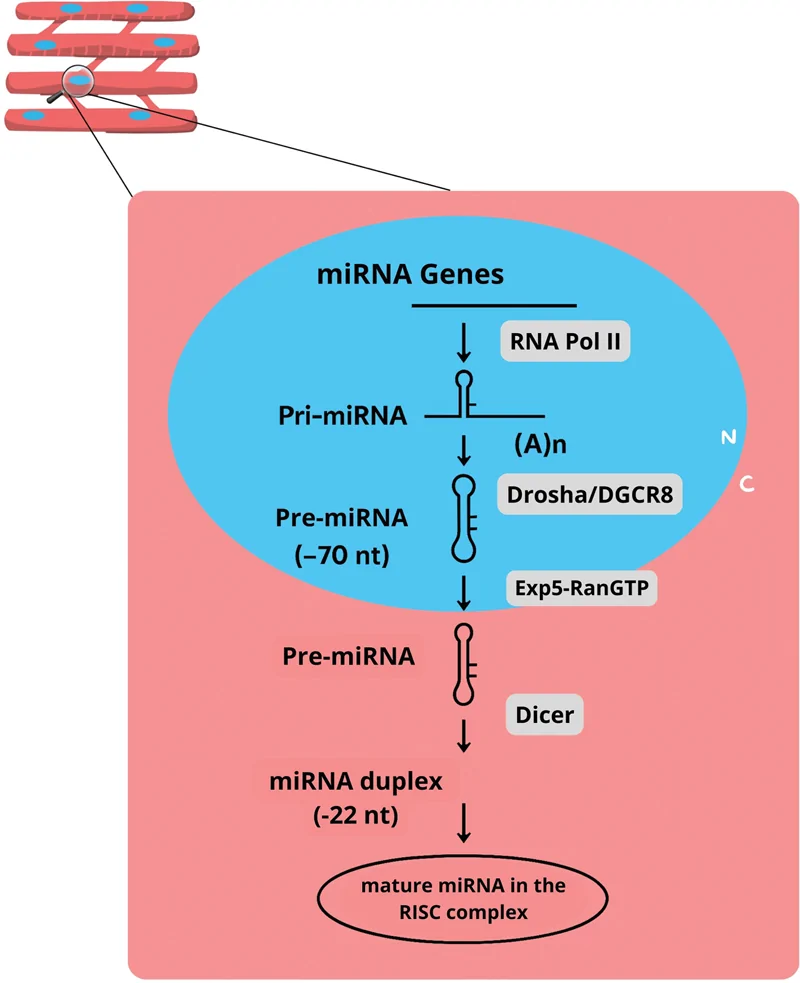
miRNA biogenesis pathway. Schematic representation of pri-miRNA transcription, nuclear processing by Drosha/DGCR8, export via Exportin-5, cytoplasmic cleavage by Dicer, and incorporation into the RISC complex.

### Gene regulation mechanisms of miRNAs

MiRNA-mediated gene regulation occurs predominantly at the post-transcriptional level. In animals, miRNAs typically bind to partially complementary sequences within the 3′ UTR of target mRNAs, with recognition largely determined by the seed sequence. This imperfect base pairing allows a single miRNA to regulate multiple target transcripts, while individual mRNAs may be targeted by several miRNAs, forming complex regulatory networks. Upon binding, the miRNA-RISC complex induces gene silencing through multiple mechanisms, including translational repression, deadenylation, and subsequent mRNA destabilization and degradation. In contrast, in plants, miRNA–mRNA interactions are often characterized by near-perfect complementarity, leading predominantly to direct endonucleolytic cleavage of the target transcript. 19 20

### Muscle-specific miRNA (myomiR) functions

Some miRNAs are widely expressed in various tissues and cells throughout the body, while others are restricted to specific tissues. Muscle-specific miRNAs (myomiRs) are a subtype of miRNAs found in muscle tissue that play essential roles in the development, differentiation, and regeneration of the muscular system. Changes in the expression of these myomiRs have been associated with several muscular dystrophies and myopathies and can be used as markers for these diseases, making them potential therapeutic targets. 21 22

Experimental research has explored the use of antagomiRs, which are molecules that block the action of harmful miRNAs and miRNA mimics, which have beneficial effects. Examples include miR-29b, which has been implicated in skeletal muscle atrophy, and miR-486, a muscle-enriched miRNA that contributes to muscle remodeling by modulating gene-expression programs associated with dystrophic muscle pathology 23 24 ( Table 1 ).

**Table 1:** MyomiR functions

MyomiRs	Functions
**miR-223–3p**	Associated with inflammatory responses in damaged/dystrophic muscle. 33
**miR-1**	Regulates muscle differentiation and contributes to skeletal muscle regeneration. 22 32
**miR-29b**	Involved in muscle atrophy. 23
**miR-31**	Modulates dystrophin expression, a potential therapeutic target in DMD. 35
**miR-133a/b**	Regulates muscle cell proliferation. 22 32
**miR-143–3p**	Increased in the serum of LGMD2A patients; potentially associated with muscle regeneration and disease-related muscle remodeling. 46
**miR-206**	Promotes muscle regeneration and satellite cell differentiation. 22 34
**miR-208b**	Associated with myosin-related gene expression and muscle fiber-type regulation. 36
**miR-486**	Muscle-enriched miRNA involved in muscle function and disease-related remodeling by modulating gene expression in dystrophic muscle. 24
**miR-499**	Associated with slow-fiber identity and mitochondrial oxidative metabolism through the miR-499/Fnip1/AMPK circuit. 36

Abbreviations: DMD, Duchenne muscular dystrophy; LGMD2A, autosomal recessive limb-girdle muscular dystrophy type 2A; miR, microRNA; MyomiR, muscle-specific microRNA.

## MYOMIRS IN MUSCULAR DYSTROPHIES AND MYOPATHIES

### Duchenne muscular dystrophy (DMD)

Duchenne muscular dystrophy is the most common and severe form of hereditary myopathy in childhood, affecting one in every 3,500 to 5,000 male births. It is an X-linked recessive disorder caused by deletions or pathogenic variants in the DMD gene (OMIM: 300377), which is responsible for encoding the dystrophin protein. This protein is essential for providing structural support to the sarcolemma during muscle contraction. The absence of dystrophin leads to membrane instability, calcium influx, cell necrosis, and the activation of inflammatory and fibrotic pathways. This results in the progressive degeneration of not only skeletal muscle, but also cardiac and respiratory tissues. Becker muscular dystrophy resembles DMD in its cause; however, it manifests as a less severe condition due to the presence of a semi-functional mutated dystrophin protein. 25 26 27 28

MyomiRs play a crucial role in regulating muscle physiology and are involved in the pathogenesis of DMD. Changes in the expression of these molecules have been associated with disease progression, making them not only promising biomarkers but also therapeutic targets. 26 29 30 31

The myomiRs described to date are: miR-1, miR-133a and miR-133b, miR-206, miR-208b, miR-499, miR-223–3p, and miR-31. These myomiRs are released into circulation in response to muscle damage and are detectable even in the early stages of the disease. MicroRNA-1, miR-133a, and miR-133b are very active during muscle formation. MicroRNA-1 promotes myogenic differentiation, whereas miR-133a and miR-133b stimulate myoblast proliferation, partly through targets such as SRF. 32 In DMD, elevated circulating levels of muscle-enriched miRNAs, including miR-1, miR-133a/b, and miR-206, have been reported and likely reflect ongoing cycles of muscle degeneration and regeneration, with some studies suggesting associations with clinical disease severity. 29 30 31 33

Another well-studied myomiR in the context of DMD is miR-206. It is induced following muscle injury and promotes satellite cell differentiation. In mouse models of DMD, an increase in miR-206 appears to be the muscle's attempt to regenerate damaged fibers. However, this regenerative response is insufficient to halt disease progression or restore muscle function in the long term. 34 Of particular therapeutic interest, miR-31 directly inhibits dystrophin mRNA translation. In some patients with pathogenic variants that permit partial dystrophin production, blocking miR-31 may enhance residual dystrophin expression, improving muscle function. 35

In turn, miR-208b and miR-499 have been implicated in muscle fiber-type regulation and myosin-related gene programs. 30 36 miR-499, in particular, has been linked to the coordination of slow-twitch fiber identity and mitochondrial oxidative metabolism through the miR-499/Fnip1/AMPK pathway. 36 In DMD, increased circulating levels of miR-208b and miR-499 have been reported, suggesting that these myomiRs may reflect disease-related muscle remodeling, regenerative activity, and changes in fiber composition rather than serving as specific markers of cardiac damage. 30

Despite the consistent upregulation of several myomiRs in DMD, important limitations must be considered. Most studies are based on small cohorts or animal models, which may not fully capture the heterogeneity of human disease. In addition, circulating myomiR levels may be influenced by factors such as physical activity, muscle mass, disease st age, and concomitant treatments, limiting their specificity as biomarkers. Furthermore, although associations between myomiRs and disease severity have been reported, causality remains unclear, and it is not yet established whether these molecules actively drive disease progression or merely reflect ongoing muscle damage and regeneration.

### Metabolic myopathy (glycogen storage disease type V)

Glycogen storage diseases are a heterogeneous group of inherited metabolic disorders characterized by defects in glycogen synthesis or degradation. These defects result in the accumulation or inadequate utilization of glycogen. McArdle disease (glycogen storage disease type V) is an inherited metabolic myopathy with autosomal recessive inheritance and the second most common glycogen storage disease, caused by pathogenic variants in the *PYGM* gene (OMIM: 608455). This gene encodes the enzyme myophosphorylase, which catalyzes glycogen phosphorolysis, generating glucose-1-phosphate. Deficiency of this enzyme compromises the degradation of muscle glycogen. Symptoms include myalgia, fatigue, muscle contractures, exercise intolerance, rhabdomyolysis and myoglobinuria, which can progress to acute renal failure in severe cases. Elevated creatine kinase (CK) levels are always present, both at rest and after physical exertion. 37

In a first-time study, Carvalho et al. 38 investigated the expression of the *PYGM* gene mRNA in Brazilian patients with McArdle disease, correlating these data with clinical, genetic, and morphological characteristics. The study examined 15 patients with confirmed diagnosis and found that the majority showed reduced mRNA expression. The authors suggest that this reduction is related to a mechanism known as nonsense-mediated mRNA decay (NMD). This process degrades defective transcripts before translation, which could explain the absence of the enzyme in the muscles. Additionally, the study showed that there was no statistically significant correlation between mRNA levels and disease severity. This suggests that mechanisms beyond *PYGM* mRNA abundance, including post-transcriptional regulation, epigenetic modulation, and alterations in protein stability or function, may influence phenotypic expression. In this context, yet-uncharacterized miRNA-mediated regulatory mechanisms could represent an additional layer of disease modulation and a promising avenue for future investigation. While in other muscle diseases, such as DMD, myomiRs have been widely described as modulators of regeneration, inflammation, energy metabolism, and tissue remodeling, in McArdle disease, this regulatory dimension remains unexplored, which limits progress in the knowledge of potential diagnostic biomarkers or therapeutic targets. 38

Taken together, the available evidence in McArdle disease supports the relevance of *PYGM* mRNA expression and post-transcriptional regulation as potential contributors to phenotypic variability. However, direct evidence regarding myomiR dysregulation in McArdle disease remains scarce, and no validated miRNA signature has yet been established for diagnosis, monitoring, or therapeutic targeting in this condition. Future studies evaluating circulating and muscle-specific miRNAs in genetically confirmed cohorts are needed to determine whether miRNA-mediated mechanisms contribute to disease modulation beyond *PYGM* transcript abundance.

### Myotonic dystrophy type 1 (DM1)

Myotonic dystrophy type 1 is a multisystemic genetic disorder with autosomal dominant inheritance that primarily affects the muscles. It results from the uncontrolled repetition of cytosine, thymine, and guanine (CTG) nucleotides in the *DMPK* gene (OMIM: 605377), which leads to the transcript gaining a toxic function. Clinically, it is characterized by muscle weakness and atrophy, predominantly in the distal regions, as well as the presence of the myotonic phenomenon, which is characterized by difficulty relaxing after muscle contraction. 39

Several myomiRs are dysregulated in patients with DM1. Muscle-specific miRNAs such as miR-1, miR-133a, and miR-133b, which play essential roles in muscle differentiation and development, have been reported to be downregulated in affected individuals, potentially contributing to impaired muscle maturation and function. 40 41 42 Conversely, miR-206 is frequently found to be upregulated in DM1 and is known to promote muscle regeneration, likely reflecting a compensatory response to ongoing muscle damage. 39 43

Importantly, circulating myomiRs have been detected in the blood of patients with DM1, supporting their potential use as minimally-invasive biomarkers for disease diagnosis and monitoring. However, variability across studies and the influence of disease stage and other clinical factors remain important limitations. 44 Although therapeutic modulation of miRNAs has been explored experimentally in muscle diseases, its application to DM1 remains investigational. Therefore, in DM1, the current evidence supports myomiRs primarily as potential biomarkers and disease-associated molecular markers rather than established therapeutic targets.

Despite these findings, the interpretation of myomiR dysregulation in DM1 remains challenging. The observed changes may reflect secondary effects of muscle degeneration rather than disease-specific molecular mechanisms. Additionally, inconsistencies across studies regarding the direction and magnitude of miRNA expression changes highlight the need for standardized methodologies. Without such standardization, the clinical applicability of myomiRs as biomarkers in DM1 remains uncertain.

### Limb-girdle muscular dystrophy (LGMD)

Limb-girdle muscular dystrophy comprises a heterogeneous group of genetic disorders characterized by progressive weakness of the proximal muscles and comprises two main subtypes based on inheritance pattern: autosomal recessive (LGMD-AR) and autosomal dominant (LGMD-AD). Each of these subtypes contains varied forms according to the affected protein involved in the structural integrity of the muscle fiber, such as calpain-3 ( *CAPN3* ; OMIM: 114240), dysferlin ( *DYSF* ; OMIM: 603009), sarcoglycans, caveolin, etc. 45

A recent study identified a distinct serum miRNA signature in patients with LGMD type 2A compared with healthy controls. In this cohort, miR-143–3p levels were significantly increased, whereas miR-486–3p levels were significantly decreased. These findings suggest that both miRNAs may serve as circulating biomarkers associated with autosomal recessive limb-girdle muscular dystrophy type 2A (LGMD2A), although their opposite directions of change indicate that they should not be interpreted simply as part of a uniform upregulated regenerative response. Notably, miR-143–3p has been linked to muscle regeneration, while miR-486–3p is a muscle-enriched miRNA involved in muscle cell differentiation, supporting the potential relevance of these molecules in disease-related muscle remodeling in LGMD2A. 46

Furthermore, several other myomiRs that are commonly dysregulated in different muscular dystrophies and myopathies, such as miR-1, miR-133a and miR-206, have also been reported to exhibit altered expression in LGMD. For example, an increase in miR-206 has been interpreted as a compensatory attempt to repair muscle fibers, as it is associated with satellite cell activation and muscle regeneration. Similarly, dysregulation of miR-133a, which regulates myoblast proliferation and differentiation, has been linked to impaired regenerative capacity. 46 47

Given that myomiRs are stable and detectable in circulation, they are being increasingly investigated as potential non-invasive biomarkers for early diagnosis, disease monitoring, and prognosis in muscular dystrophies. The specific expression profile of circulating myomiRs may also contribute to differentiating between LGMD subtypes and complement genetic findings. However, the available evidence remains restricted to a small number of studies, often focused on specific subtypes, which limits the generalization of these findings across the broader LGMD spectrum. 47

Therefore, similar to other muscular dystrophies and myopathies, the specificity of these myomiRs remains limited, as many are commonly dysregulated across different muscle diseases. This overlap complicates their use in differential diagnosis. In addition, most studies focus on specific LGMD subtypes, making it difficult to extrapolate findings to the broader LGMD spectrum. Larger, multicenter studies are needed to validate these biomarkers and determine their clinical utility.

### Myofibrillar myopathy (MFM)

Myofibrillar myopathy (MFM) is a heterogeneous group of hereditary muscle diseases, predominantly transmitted in an autosomal dominant pattern, although rare autosomal recessive or X-linked forms have also been described. It is characterized by dissolution of myofibrils, the presence of intracellular protein aggregates (often involving Z-disc or sarcomere proteins such as desmin, αB-crystallin, myotilin, filamin C), and structural changes in muscle fibers. Clinically, patients present with progressive muscle weakness, which may involve proximal and/or distal muscles, with possible cardiac and respiratory involvement. 48 49

Published evidence specifically addressing myomiR dysregulation in myofibrillar myopathy remains very limited. Therefore, the available information should be interpreted as preliminary and hypothesis-generating rather than as established evidence. Preliminary data from an unpublished study suggest altered expression patterns of miR-133a, miR-133b, miR-499, miR-208a and miR-486 in muscle tissue from patients with MFM compared with controls. This suggests that these post-transcriptional regulators are involved in the pathophysiology of the disease. RT-qPCR analysis of biopsy samples from 16 patients with MFM and 18 controls indicated a significant increase in the expression of miR-133b and miR-499 in the MFM group. In contrast, miR-208a expression was higher in the control group. Furthermore, relevant changes and statistically significant correlations were observed in the expression of miR-133a and miR-486, as well as with the expression of structural genes. Concomitantly, mRNA levels of classic MFM-associated genes ( *FLNC* , *DES* , and *ZASP* ) were significantly upregulated ( *p* < 0.0001), while *MYOT* (OMIM: 604103) remained unchanged. This suggests that transcriptional alterations of sarcomeric proteins could coexist with disruptions in miRNA regulation. 50

Specific correlations were identified, such as the negative association between miR-133a and *ZASP* / *FLNC* mRNAs in the *MYOT* mutation subgroup, and the reversal of the correlation between miR-486 and *ZASP* (positive in controls and negative in *ZASP* mutation patients). These findings indicate that distinct genetic variants can differentially modulate myomiR-mRNA networks, resulting in a loss of fine regulation of the Z disc and compensatory or toxic gain-of-function responses. These patterns could explain the hypothesis that myomiRs are involved as both markers of the degenerative/remodeling process and modulatory effectors of sarcomeric repair and homeostasis pathways. This reinforces their potential as diagnostic biomarkers and mechanistic targets. However, these findings should be interpreted with caution, as they are derived from unpublished data and have not yet undergone peer review. In addition, the small sample size and genetic heterogeneity of the cohort further limit the robustness and generalizability of these observations. Therefore, independent validation in larger, well-characterized populations is essential before any definitive conclusions can be drawn regarding the role of myomiRs in MFM 50 ( Table 2 ).

**Table 2:** Summary table of myomiRs versus muscular dystrophies and myopathies

MyomiRs	Reported disorders	DMD	McArdle disease	DM1	LGMD	MFM
**miR-1**	DMD > DM1 > LGMD	↑	−	↓	*	−
**miR-29b**	DM1	↓	−	↓	−	−
**miR-31**	DMD	↑	−	−	−	−
**miR-133a**	DMD > DM1 > LGMD	↑	−	↓	*	*
**miR-133b**	DMD	↑	−	−	−	↑
**miR-143–3p**	LGMD	−	−	−	↑	−
**miR-206**	DMD > DM1 > LGMD	↑	−	↑	↑	−
**miR-208b**	DMD	↑	−	−	*	−
**miR-486**	LGMD	−	−	−	↓	*
**miR-499**	DMD > LGMD	↑	−	−	*	↑

Abbreviations: DMD, Duchenne muscular dystrophy; LGMD, limb-girdle muscular dystrophy; MFM, myofibrillar myopathy; miR, microRNA; MyomiR, muscle-specific microRNA.

Notes: ↑: up-regulated; ↓: down-regulated; *: dysregulated; -: information not cited.

## CURRENT LIMITATIONS AND FUTURE PROSPECTS

Despite the growing interest in myomiRs as potential biomarkers for muscular dystrophies and myopathies, important limitations in literature remain to be overcome. One of the main issues is the lack of standardization between studies, with different studies using different methods to collect, process, and analyze myomiRs. This makes it difficult to compare results and draw solid conclusions. Additionally, most studies have been conducted in laboratories or with small patient cohorts, with a dearth of clinical studies in humans. While the initial results are promising, it is still too early to apply these findings broadly in medical practice.

However, with more clinical research, standardized methods and the increasing use of new technologies, myomiRs may soon become valuable tools in the care of patients with muscular dystrophies and myopathies. This could facilitate earlier diagnosis, patient stratification, and treatment monitoring in clinical practice.

In conclusion, myomiRs have emerged as important regulators of the molecular pathways underlying muscle degeneration and regeneration in muscular dystrophies and myopathies. The evidence reviewed highlights their involvement in key pathogenic processes, including inflammation, fibrosis, and impaired muscle repair, particularly in well-characterized conditions such as DMD. In addition, their stability in biological fluids supports their potential as minimally invasive biomarkers for disease detection and monitoring.

However, despite these advances, their clinical applicability remains limited. The current body of evidence is heterogeneous across different muscular dystrophies and myopathies and is still largely based on small cohorts and preclinical models. Furthermore, the lack of disease specificity and variability across studies represent important barriers to their implementation in clinical practice.

As discussed above, overcoming these challenges will require methodological standardization, validation in large and well-characterized populations, and a deeper understanding of the functional role of myomiRs in disease pathogenesis.

Overall, while myomiRs represent a promising tool for improving the diagnosis and monitoring of muscular dystrophies and myopathies, their translation into routine clinical use will depend on addressing these current limitations.
